# An Evolutionary-Based Framework for Analyzing Mold and Dampness-Associated Symptoms in DMHS

**DOI:** 10.3389/fimmu.2016.00672

**Published:** 2017-01-09

**Authors:** Alvaro Daschner

**Affiliations:** ^1^Instituto de Investigación Sanitaria, Hospital Universitario de la Princesa, Servicio de Alergia, Madrid, Spain

**Keywords:** evolution, allergy disease, symptom perception, behavior, IgE

## Abstract

Among potential environmental harmful factors, fungi deserve special consideration. Their intrinsic ability to actively germinate or infect host tissues might determine a prominent trigger in host defense mechanisms. With the appearance of fungi in evolutionary history, other organisms had to evolve strategies to recognize and cope with them. Existing controversies around dampness and mold hypersensitivity syndrome (DMHS) can be due to the great variability of clinical symptoms but also of possible eliciting factors associated with mold and dampness. An hypothesis is presented, where an evolutionary analysis of the different response patterns seen in DMHS is able to explain the existing variability of disease patterns. Classical interpretation of immune responses and symptoms are addressed within the field of pathophysiology. The presented evolutionary analysis seeks for the ultimate causes of the vast array of symptoms in DMHS. Symptoms can be interpreted as induced by direct (toxic) actions of spores, mycotoxins, or other fungal metabolites, or on the other side by the host-initiated response, which aims to counterbalance and fight off potentially deleterious effects or fungal infection. Further, individual susceptibility of immune reactions can confer an exaggerated response, and magnified symptoms are then explained in terms of immunopathology. IgE-mediated allergy fits well in this scenario, where individuals with an atopic predisposition suffer from an exaggerated response to mold exposure, but studies addressing why such responses have evolved and if they could be advantageous are scarce. Human history is plenty of plagues and diseases connected with mold exposure, which could explain vulnerability to mold allergy. Likewise, multiorgan symptoms in DMHS are analyzed for its possible adaptive role not only in the defense of an active infection, but also as evolved mechanisms for avoidance of potentially harmful environments in an evolutionary past or present setting.

## Introduction

After several decades of research in the field, the systemic health effects of mold exposure still seem to be a controversial issue. Several mainly methodological reasons account for this scenario. Fungal spores and components, unlike other bioaerosol particles, are more heterogeneous and biologically dynamic particles. Scientific advances have provided us with some analytic procedures to estimate the environmental load of fungi by measuring β-glucan or *N*-acetylhexosaminidase (1, 2) and airborne fungal cell fragments (3), and by assessing the presence of mycotoxins in the environment or even in human samples (4–6). Further, molecular biology is able to detect fungal DNA in human samples or tissues, such as in sinus (7). Measurements of fungal cell agents are more precise than ocular inspection to detect fungi (1, 8). Nevertheless, these possible biomarkers are not being widely used for research; epidemiologic studies or the clinical practice and reality is that exposure assessments are often based on subjective methods (9). In the case of inflammatory markers associated with mold exposure and clinical health effects, the difficulty for using them arises because these inflammatory markers are frequently unspecific in nature and lack sufficient evidence for causality, whereas epidemiologic studies need specific biomarkers of both mold exposure and clinical involvement. In the habitual clinical setting, the specialist commonly uses only biomarkers of disease and uses the clinical history for estimation of exposure to noxious agents. In fact, in allergology, the clinical history still represents the most important criterion when assessing a patient. Positive tests have to be in accordance with a plausible relationship between the patient’s complaints and his exposure history in order to avoid false-positive associations. These results are not only due to possible irrelevant cross-reactivity or methodological issues but also because the presence of IgE is not a necessary indicator of mold exposure (10). A major problem is on the other side, the lack of measurable scientific evidence, when patients and doctors see a plausible correlation of mold exposure and attributed health problems without having specific biomarkers of exposure, which support the claimed interrelation. Most clinical studies have used self-reported symptoms and were based on subjective complaints prone to bias and confounders (10). Finally, even if IgE could in some instances serve as a valuable biomarker of effect as evidenced in mold allergy, a recognition pattern of allergens resulting from individual exposure and sensitization will always be patient specific, not only because of interindividual’s varying exposure histories but also genetically determined constraints of recognition of immunogens. The recognition patterns of allergens resulting from exposure to complex allergenic sources are always patient specific (11).

**Figure 1:**
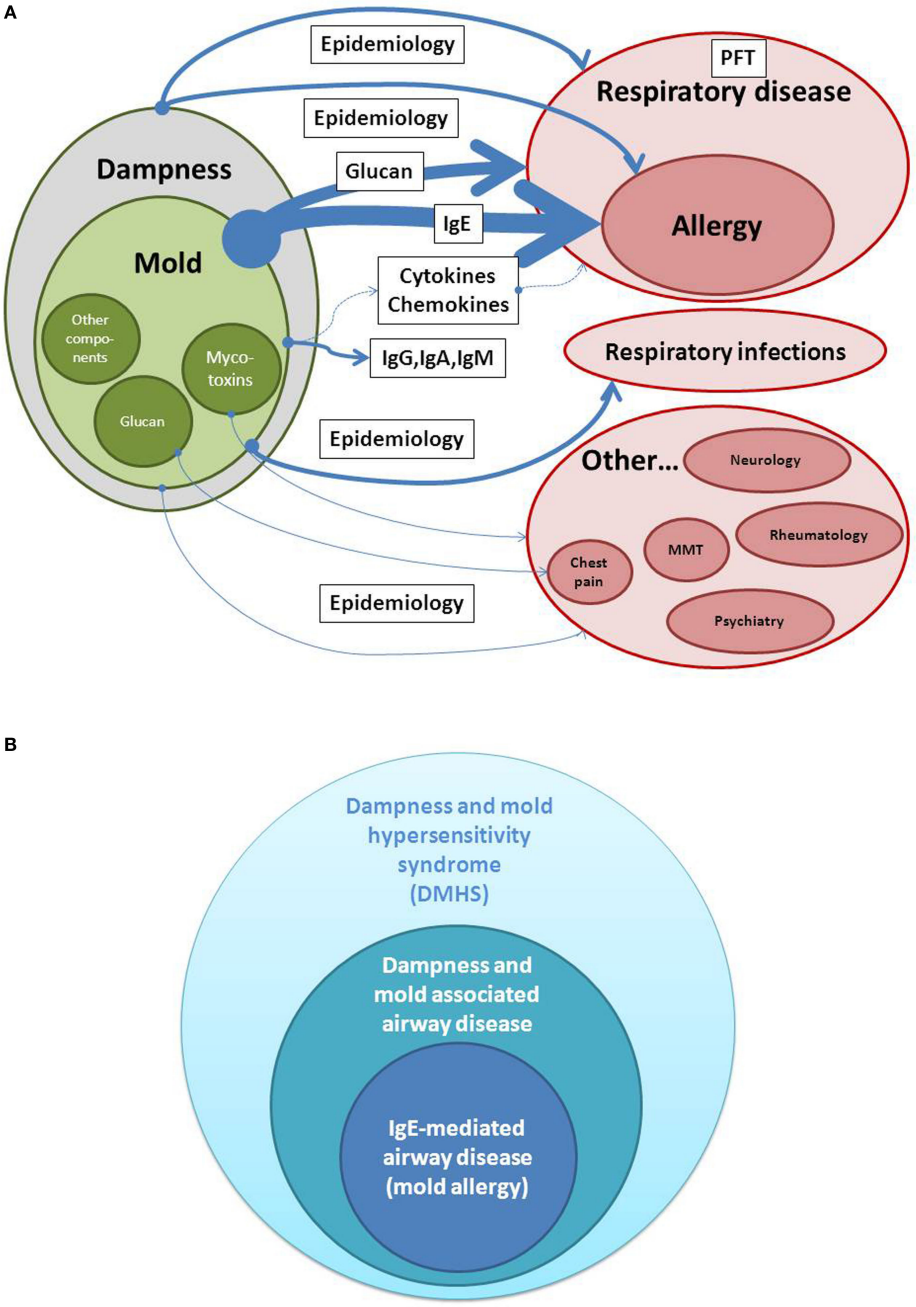
**(A,B)** Estimates of evidence for a causal relationship between dampness/mold exposure and disease. Whereas detection of IgE is used as a biomarker in allergic airway disease and belongs to the highest evidence in dampness and mold hypersensitivity syndrome (DMHS), extra-respiratory symptoms are less consistent when analyzed in different epidemiologic studies. PFT, Pulmonary function testing; MMT, Mixed mold mycotoxicosis.

Figure 1A shows estimates of evidence for the causal relationship between dampness and mold exposure in buildings and health effects. It shows that the relationship between dampness and mold and respiratory disease is mainly clinical and epidemiological, and besides IgE as a biomarker of allergy, other biomarkers of exposure or specific of mold-induced disease are beginning to be recognized only now. This explains that visible mold or dampness is frequently assessed as an environmental factor and in fact correlation with clinical health effects is frequently higher than when specific exposure markers have been used in distinct settings (12). The presence of visible mold serves as a good indicator that the indoor environment is out of balance (13). Damp housing conditions are repeatedly reported to be associated with respiratory illness both in atopics and in non-atopics and both in public and in domestic environments (14–16). In these damp housing conditions, there are some indications that mold spores were associated with the symptoms (16). Within dampness and mold hypersensitivity syndrome (DMHS), the connection between mold exposure and allergic respiratory disease belongs to the most evident relationships (see below).

Moving from diseases with accepted biomarkers and more defined nature of symptoms, such as respiratory allergy, to syndromes or symptoms, which are frequently subjective in nature, such as cognitive difficulties, memory loss, or fatigue, complicate the evaluation of cause and effect relationships in DMHS (Figure 1B). Therefore, in the last decades, different causal and syndromic relationships have the description of “sick building syndrome,” “mixed mold mycotoxicosis,” or other environmental sensitivities. Even chronic fatigue syndrome or clinical features similar to this entity have been connected not only to relevant environmental histories of exposure to water-damaged buildings but also to the detection of mycotoxins (4). These descriptions frequently overlap with other diseases in an autoimmune context.

The study of causality in epidemiology or toxicology is not only characterized by difficulties when assessing monocausality or multicausality or a specific syndrome or disease. Difficulties also arise by the complex mixture of health outcomes with exposure to the same suspected agents ranging from frequently asymptomatic subjects to clear-cut allergic or non-allergic respiratory disease to various accompanying symptoms in several systems. Peoples’ responses to factors in their environment vary enormously (17). Discrepant symptoms from equally exposed subjects can be due to differences in susceptibility in genetically determined biochemical and immunological traits and thus these are the reasons for contradictory results or negative results in clinical studies. There is also a tendency to misinterpret the lack of evidence for causation as evidence for lack of a causal relation. In this sense, causality criteria can be used to falsely postpone public health action under the pretext that the available evidence does not fulfill the criteria (18).

Evolutionary medicine is the application of modern evolutionary theory to the understanding of health and disease. It searches for evolutionary explanations of disease and symptoms as complement to the analysis of the proximate causes of disease (pathophysiology). Analyzing the complex relationship between mold exposure and health effects by evolutionary theory could help to explain the existing controversies and inconsistencies in research findings.

I will show that the existing controversies around DMHS due to the great variability of clinical symptoms and also of possible eliciting factors associated with mold and dampness account for the methodological constraints of clinical studies in DMHS and eventually to lack of consistent findings if an evolutionary approach is not used. An evolutionary analysis of the different disease patterns seen in DMHS is able to explain these, mainly as an attempt to avoid and defend exposure to potential harmful noxious agents.

## Fungi in an Evolutionary Perspective

For an evolutionary analysis of DMHS, we should first investigate the ecological relationship between humans and molds, the possible coevolution or absence of coevolution between mold and humans, and then analyze the relationship between immune features and the explanation of symptoms as possible defense mechanisms, not excluding the study of a neurobehavioral component.

Fungi and molds are ubiquitous and comprise as much as 25% of the world’s biomass and make up to 4–11% of fine particle mass in urban and rural air (19). Noteworthy, fungal spores are often 100–1,000 times more numerous than other bioparticles, such as pollen (20). Among potential environmental harmful agents, fungi deserve special consideration. This is also true when considering them as potential infectious agents, as they are microbes that can be acquired directly from the environment (21).

Overall, the relationship between humans and fungi denotes a paradox. There are only a few thermal dimorph species able to infect, persist, and cause disease in healthy hosts (22). Invasive fungal infections are rare, and it has been noted that humans and other mammals have a remarkable resistance to fungal pathogens (22, 23). On the other side, if fungal infection is invasive, it is among the most difficult diseases to manage and frequently lethal (22).

**Figure 2:**
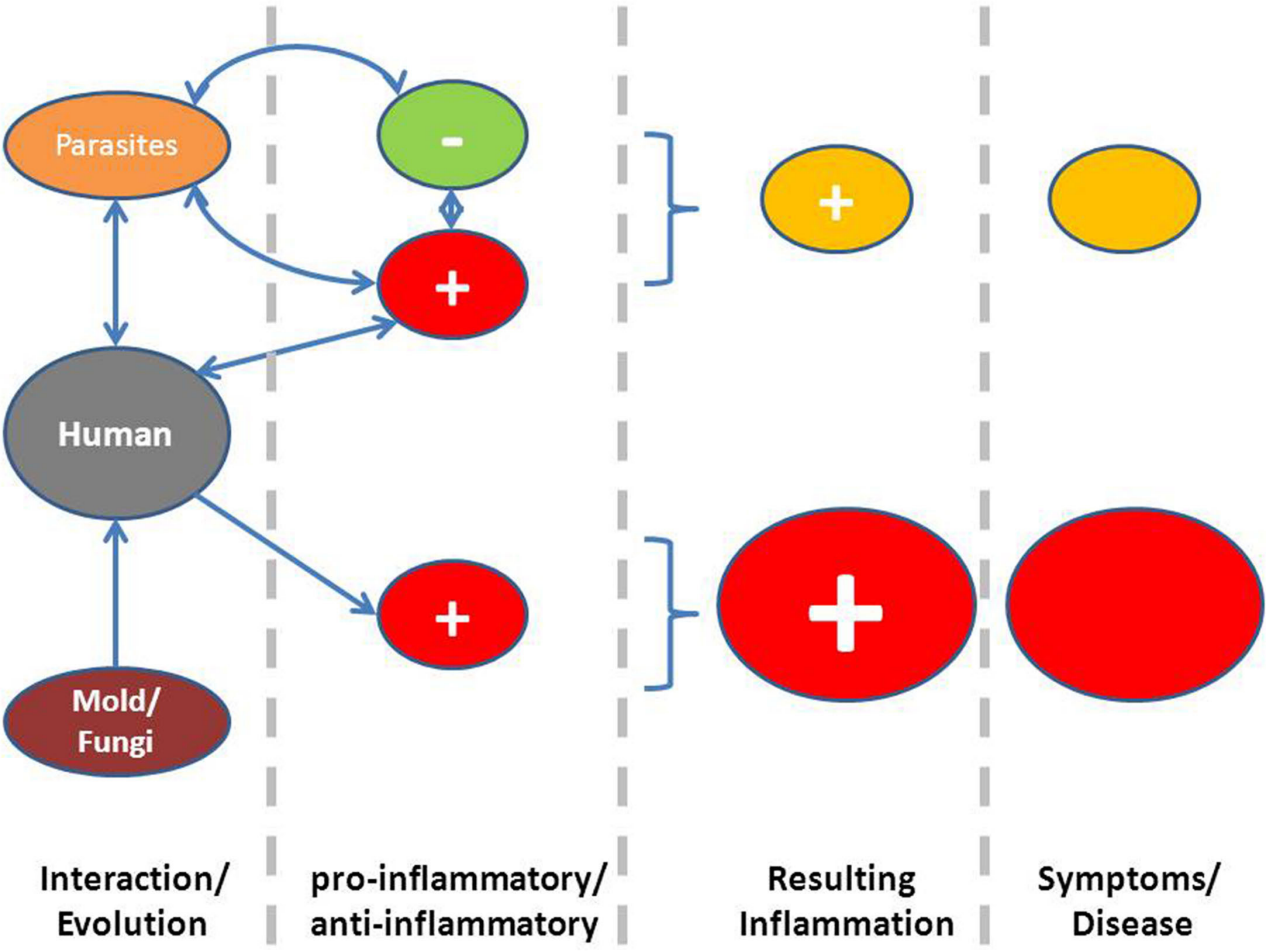
**Proposed hypothesis linking exaggerated symptoms due to a more pro-inflammatory status as a consequence of a lack of coevolution between most fungi and humans (below), whereas in the case of coevolution between parasites and humans, inflammation is downregulated by chronic infection**.

An evolutionary analysis shows that for most fungi, it is not necessary to rely on a life host for propagation. Human mycoses are seldom contagious (23). In an evolutionary host–parasite relationship as proposed for other infectious agents, such as virus, parasites, or bacteria, the outcome of parasite virulence depends on how symptoms would affect transmission probability (24). Natural selection provides a pressure on microorganisms and parasites, which depends on host to host transmission to “self-limit” virulence, inducing an equilibrium point of virulence, where parasite’s fitness is highest. The absence of a host–parasite relationship, such as between most fungi and humans, without mechanisms of coevolution, is eventually associated with higher pathogenicity. A higher investment in prevention and elimination of these potential invaders by the host is therefore to be expected, such as an important pro-inflammatory component of the host response (Figure 2).

Fungi appeared soon in the evolutionary history of life and other organisms had since then to evolve strategies to recognize and cope with them. Classical examples of deleterious effects of mold or mycotoxins come mainly from the field of alimentary toxins, such as neutropenia associated with tricothecenes produced by *Fusarium tricinctum*, other immunomodulatory effects of fungi or the effect of ergotism throughout human history by *Claviceps purpurea* (25). Another example is the recent discovery of fungal cells and hyphae in the tissue of the central nervous system of patients with Alzheimer’s disease, which is not found in control subjects (26). Other neurodegenerative diseases, such as amyothrophic lateral sclerosis or multiple sclerosis, have been associated with fungal infection (27, 28). Estimates have shown that more people die from fungal infections than from tuberculosis or malaria together (29). Modern way of life leads people to spend more than 80% of their time indoors, but at least as late as in Antiquity (Leviticus), fungal infestation of buildings has also been mentioned.

Notwithstanding, fungi could have played an important role in selecting for traits able to avoid deleterious effects of fungi, mold, and their metabolites. Endothermic and homeothermic lifestyle of mammals has been suspected to account for the restricted pathogenicity of the vast number of fungal species (21). Invasive fungal disease is rare and appears mainly in patients with impaired immune function. But there are also case reports describing invasive fungal disease, such as invasive sinusitis or rhinosinusitis (30, 31) or craniocerebral aspergillosis of sinonasal origin, in immunocompetent patients (32). These data tell us that the powerful immune defense system of humans has in fact evolved effective mechanisms to avoid ubiquitous fungi to be pathogenic in most immune-competent subjects, but the arms race between fungi and the immune system of vertebrates is still visible.

## Hypothesis

How can an evolutionary approach help to disentangle the apparent lack of proof for mold- and dampness-associated health effects? The following approach is not intended to strengthen the evidence of DMHS, but can be helpful to explain inconsistencies when analyzing different study results and could be useful to the design of future studies. The evolution of causality in epidemiology has changed over the last century from a monocausal approach to a polycausal understanding (33), which should not hinder to analyze possible effects of dampness *per se*, or mold and/or its different disease-causing components, such as volatile organic compounds, hyphae, spores, and mycotoxins. But the possible drawback in DMHS is that clinical symptoms are varying in the syndrome and many claimed that often non-specific health complaints have been described, ranging from respiratory to rheumatic, neurological, systemic, and even behavioral symptoms. An evolutionary approach would therefore be most helpful by analyzing the effect-side of the cause-effect relationship attempting to explain the vast range of symptoms in the context of interindividual variability.

I hypothesize that variability in symptoms and susceptibility to mold and dampness can be explained by analyzing the relationship between fungi, mold, and humans over evolutionary time, its impact on the immune and behavioral system, and the resulting visible defense mechanisms as disease. No previous hypothesis has been published on the possible evolutionary origins of DMHS, as the main focus in studies addressing causality of symptoms and disease associated with exposure to water-damaged buildings has been the search for proximate causes in a pathophysiological evaluation, such as the toxic effects of mycotoxins, the mechanism of hypersensitivity symptoms in IgE-mediated reactions, or the analysis of multiorgan complaints. An ultimate causation analysis highlights the biological origins of the different response patterns, selected over evolutionary timescales because of putative adaptive values in the past and possible adaptive values in the present.

A central evolutionary hypothesis states that symptoms associated with mold exposure can be analyzed with respect to a possible adaptive function. This does not necessarily mean that they are adaptive in a concrete individual but that the diversity of response mechanisms and the preponderance of apparently exaggerated symptoms could be due to the evolutionary history of humans and its relationship with exposure to fungi and their metabolites (see also Box 1). Exposure to fungi is not only associated with unspecific and specific host defense mechanisms but probably also with a capacity to induce a more pro-inflammatory immune response and lack of anti-inflammatory regulation, such as it is known with microorganisms or parasites (Figure 2). Further, important disease avoidance mechanisms can explain frequent symptoms induced by reflexive or signaling mechanisms, when these are able to inform the individual to avoid exposure or re-exposure to a potentially damaging environment. This also explains that not only the presence of different molds or fungi is able to induce symptoms, but associated factors, such as dampness *per se*, could also induce symptoms. If symptoms are mainly due to the host defense arm in the interaction between fungi and humans, it also explains interindividual variability in defense-associated symptoms or behavioral patterns as well as a wide range of possible eliciting factors, which can include olfactory and visual signals.

Box 1 **Unnecessary or exaggerated symptoms.**

Evolutionary medicine takes into account a trade-off model, where different adaptations to changing environments are associated with disease susceptibility. After ruling out that symptoms are produced by direct toxic effect (difficult in the clinical practice) and when considering them as a defense mechanism by the host, the following items have to be taken into account: frequently, symptoms are exaggerated and the defense amplitude is not correlated with the necessary response. This can also be due to the smoke detector principle with expression of a false alarm, when the cost of expressing a defense is low compared to the potential harm it protects against (52). Therefore, it is to be expected that the defense reaction is able to produce uncomfortable consequences or adverse health effects, not necessarily related to the current pathogen avoidance or defense. In our special case, the cost of fungal infection with high mortality could explain the often exaggerated symptoms and multisystem complaints in DMHS by the intrinsic selection pressure. Otherwise not all defense reactions should be considered adaptive now or postulated having been adaptive in the evolutionary past. The real potential of symptoms in DMHS to have an ongoing adaptive function can only be assessed in future studies, when latent infection or subclinical colonization by fungi, as well as severe systemic infections or other direct toxic harming effects are assessed and patients with similar exposure histories with and without MDHS are compared.

**Figure 3:**
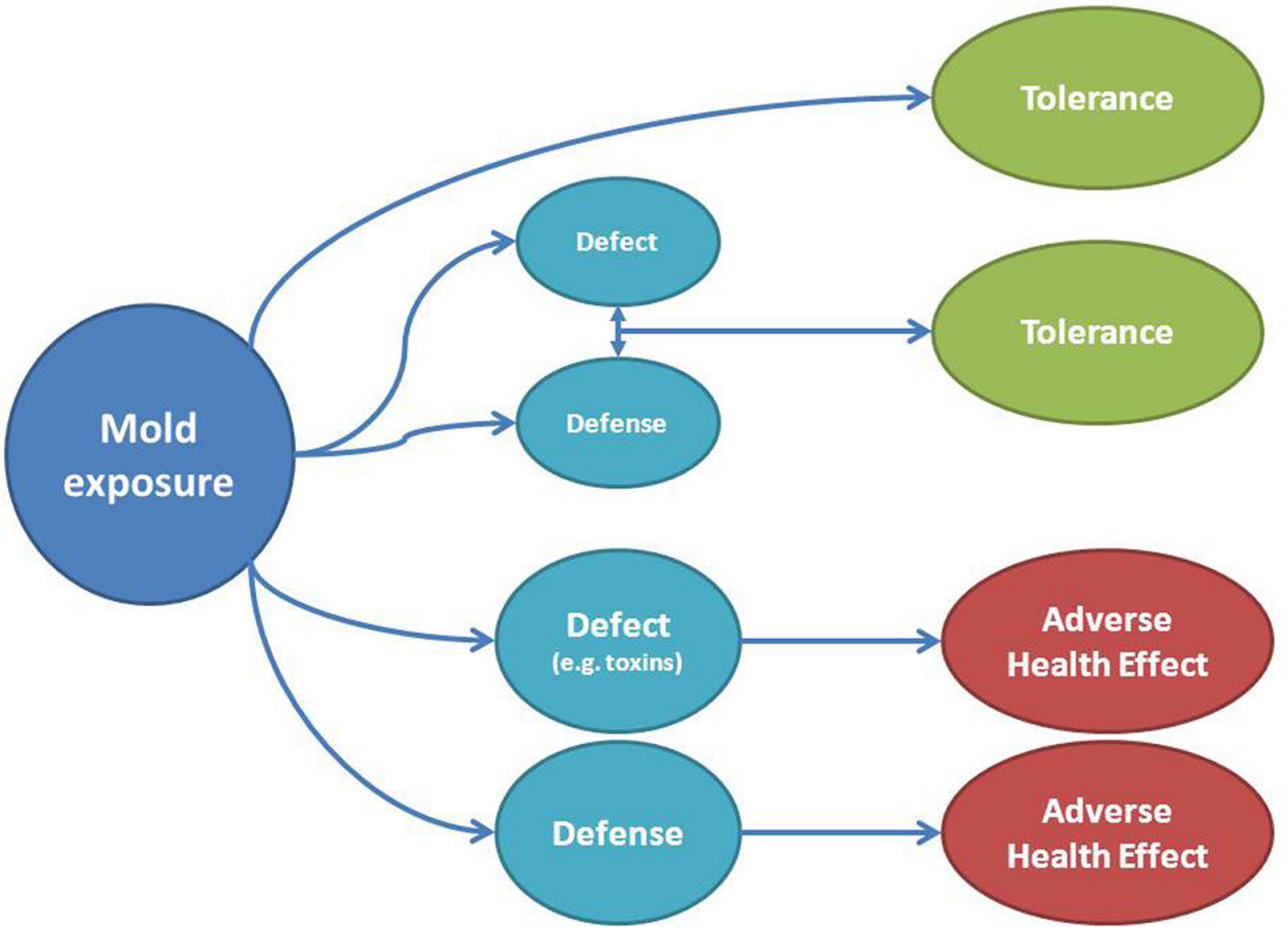
**Adverse health effects or tolerance to mold exposure depend on the relationship between defect and defense**. A toxic mechanism on the host or its organs can be directly produced by mold and be visible as adverse health effect (e.g., by toxins), but symptoms can also be elicited by a response of the host (e.g., inflammation) to a mold component. Tolerance can be due to a lack of interaction between mold and the host or by the neutralization of a possible toxin by the host response (above). Note that in the clinical setting, it can be difficult to disentangle every postulated mechanism.

Besides mold able to infect humans, other pathophysiological mechanisms of symptoms or disease can be summarized to be a toxic-irritant effect from mold metabolites or the generation of a deleterious immune response (34). When considering symptoms, these can thus be interpreted to be elicited by a direct damaging or toxic effect, by the induction of a defense response by the host or both (Figure 3). Figure 3 also shows how tolerance to the same agent can be interpreted as a lack of interaction between mold and the host, or on the other side, a potentially damaging effect is neutralized by mounting a simultaneous defense. The reader should be aware that the proposed effects cannot be completely differentiated in the real-life situation but individual susceptibility encountering the same potentially damaging agent can be explained.

As can be seen in Figure 1B, scientific evidence for DMHS is more difficult to ascertain when symptoms or implicated organs do not belong to the respiratory tract and comprise a multiple non-respiratory system syndrome, mainly because of lack of a robust objective indicator of illness and effect of mold on it. As an example of extra-respiratory symptoms, mold exposure to *Stachybotrys chartarum* has been associated with rashes, blurred vision, memory loss, headache, fatigue, nausea, balance loss, cognitive dysfunction, or even hemorrhage and seizures (35).

In the next sections, I will go through the different mechanisms of defense, which include classical immune phenomena, and also those produced by neuroendocrine pathways. I will show that these mechanisms are not only associated with the well-known “purpose” of *defending* pathogens but also of *avoiding* them as a prevention strategy. It is exactly the variability of mechanisms and thus occurrence of symptom complexes of the last feature that could explain the lack of consistent findings in clinical studies, if the biological meaning of evolved mechanisms and symptoms is not taken into account.

## Defense and Avoidance

If it is true that dangerous fungal infections have influenced our evolutionary history, natural selection would have acted on those traits, which confer less risk of infection. The overall resistance of mammals to fungal disease and the fact that most human systemic fungal disease are opportunistic in nature, and associated with important immunodeficiency should explain the existence of a vast host defense machinery to prevent infection by these organisms. Therefore, reflexive or immune-mediated symptoms associated with mold exposure before infection should be interpreted as a means to avoid the costly defense mechanisms or even reduced fitness and death, which would arise when the pathogen has already entered the host.

The first line of defense is anatomical, and mechanisms, such as muco-ciliary clearance, represent a first strategy for removal of airborne fungi (20). We should also include the microflora of saprophytic microorganisms, which compete with potential intruders to a local ecologic equilibrium (36). Further defense lines are the innate and the adaptive immune system [reviewed in Ref. (37)]. Symptoms can be induced by the inflammatory reaction, but can also arise by reflexive mechanisms. The inflammatory mediators induced by an immune response to offending agents are further able to provoke a sickness behavior, such as sleepiness, depression, apathy, and social withdrawal (38).

The defense mechanisms are not necessarily directed only against the dangerous spore or invading hyphae. Besides the commented preventive disease avoidance mechanisms, signals associated with these potential noxious agents can be toxins, secreted proteins, or even proteins or other signals associated in time or space. Some examples of host responses to indirect non-offending signals associated with dangerous factors are the following: Profet hypothesized that IgE against specific non-offending proteins could be elicited in order to avoid entry of potentially damaging toxins, when these are linked (39). Lappé hypothesized that IgE production against mites could have evolved sometime after the agrarian revolution as a tocsin or warning that a person is in an environment conductive to mold-related illness, because moldy, storage crops require humidity, and humidity encourages infestation with storage mites (40). Toxins in contaminated food can be the best sign of a pathogen’s presence (41). Extending the idea, airborne mold-associated particles, such as volatile organic compounds, can serve as a signals of potentially harmful invaders in the environment and all human sensory organs can be implicated in different mechanisms of disease avoidance. Thus, it could be summarized that defense mechanism could be initiated when certain signals are associated with a particular probability of exposure to harming fungi or molds.

Immune phenomena inducing defense against noxious agents can be induced by different means, but the final effector mechanisms are accompanied by inflammation. In fact, the mechanisms of non-allergic asthma (without IgE production) result frequently from similar inflammatory changes, and the implication of other immunoglobulin isotypes cannot be ruled out (42). Likewise fungal species induce inflammatory processes and histamine release without implication of an IgE-mediated mechanism (43).

The immunologic Th2 response to parasites has been associated with the development of effector mechanisms that help to clear host organs from multicellular parasites. Mast cells, but also eosinophils and other immune cells, contribute to the release of mediators that eventually lead to a “weep and sweep” response, which is characteristic of many intestinal helminth infections, rendering an inhospitable environment for the helminth parasite (44).

On the other side, in the case of *Aspergillus*, and also other fungi, the production of aTh1 immune response appears to be protective, whereas Th2 responses is not (37). Achieving a balance between Th1 and Th2 cytokines may be important for optimal antifungal protection while minimizing immune-mediated damage. In general, a Th1 response is required for clearance of a fungal infection, while Th2 immunity usually results in susceptibility to infection or allergic responses (45). Th1 and Th17 are the principal T helper subsets shown to contribute to protective immunity to several pathogenic fungi (46).

Visual signs or specific odors allow us to avoid putrid food, a behavior that is able to save lives and for some substances, such as geosmin, an extremely high sensitivity of the human nose has been detected (47). Disease avoidance mechanisms taken by an animal are aimed at reducing its chances of becoming infected with pathogens or parasites.

A classical infection-avoidance behavior is disgust, a possible pan-mammalian adaptation, where emesis is associated with a protective response and existence of a clear association between signal and disease (41, 48). Disgust sensitivity varies between individuals as a trait and within individuals by their state and also through individual learning or imitation (48). At the cost of a possibly high rate of false-positive detections, this mechanism survived by evolution detecting pathogen threats, mainly in food and besides bacterial contamination by sensing visual, olfactory, or gustatory signals includes foodborne disease by fungi and associated mycotoxins. Another issue is that repeated exposures to certain kinds of danger may adaptively lower response thresholds (49) and thus explain interindividual variability of symptoms due to different past exposure histories.

Given the choice, we prefer clean, dry environments in which to live (50). The explanation comes from extension of the disgust mechanism, and also from learning behavior. Unhealthy environments are thus able to elicit symptoms caused by reflexive or learned mechanisms, which in turn help to avoid this environment and a higher probability of disease by environmentally acquired pathogens. On the other side, an exaggerated or unnecessary immune response or reflexive behavior leads to unpleasant symptoms and disease. Like inflammatory disease being the trade-off for a powerful immune response against pathogens, also behavioral mechanisms have the potential for producing consequences unrelated to pathogen avoidance. Obvious difficulties arise when studying and assessing these often subjective symptoms with lack of measurable specific markers. The role of mold components, such as mycotoxins, in diseases caused by fungi growing inside buildings is controversial (51). The above reasoning could explain the controversial findings when assessing the health effects of mold components and therefore much of the symptoms could possibly be explained by an exaggerated host response and not by a direct toxic effect.

In the clinical practice, one of the problems associated with the study of these symptoms in relationship with claimed mold exposure is that patients see an attempt by physicians to take refuge in a psychiatric label (17). Further, some behavioral responses could be elicited by learned or conditioning reflexes by the attempt to avoid repeated unpleasant situations. But an evolutionary perspective can also help here to interpret psychological shortcuts or signals. Again, the lack of both widely accepted specific biomarkers and the specificity of symptoms accounts for the controversy in this study field. Whereas a classical immune reaction serves to avoid invasion or multiplication, when fungi and mold have already gained access to some organ, neuropsychiatric symptoms could be interpreted as a behavioral response “intended” to avoid further environmental exposure or as signals for the need for help and treatment to potential helpers. After ruling out direct neurotoxic effects accounting for the presence of these symptoms, some of them, such as social withdrawal or depression, can be discernible to other group members and could have evolved as defense mechanisms (38). An evolutionary-based interpretation of psychiatric disease is difficult, but in DMHS these symptoms, if present, are frequently accompanying more classical, measurable respiratory symptoms. Thus, suffering subjects, if they are aware of the relationship between possible exposure and symptoms, will try to avoid this exposure.

It is thus clear that humans, like other organisms, must have evolved mechanisms not only to cope with invading harmful pathogens, but also to avoid or reduce exposure to them in order to prevent them to gain entry into vital systems (50, 52).

## Allergic and Non-Allergic Respiratory Disease

Sneezing and cough, and also airway mucus production, are typical in respiratory infection. Virus infections damage airway epithelium producing part of the symptoms, but the local inflammatory response by the host produces the other part of the symptoms. As respiratory infections are transmitted by sneezing or by droplets, a classical way to analyze these symptoms in an evolutionary perspective is to interpret the induced response as a manipulation of the host to aid dispersal of the disease and/or a defense by the host to eliminate the disease (53).

But cough and other reflexive symptoms being unpleasant have a further explanation, in that their aversiveness motivates escape and future avoidance of situations that could have caused the response (54).

Among inhalant allergens and compared to other common allergens, such as house dust mites, dander, or pollen, the role of fungi in allergic airway disease has always been more controversial. For example, it has been asserted that fungal allergens in research and molecular allergology are largely neglected (55). This is due to a still limited power of diagnostic tools compared to other aeroallergens. The fungus kingdom is especially difficult for assessing allergy due to incompletely validated standardized extracts, difficulties in the production of allergenic molecules, and also the presence of phylogenetically conserved cross-reactive allergens (11). Commercially available extracts used for diagnosis and assessment of mold allergies do not cover the majority of molds identified in the indoor air of buildings with moisture problems (56). There are however epidemiologic data that consistently support the relationship between fungal exposure and allergic airway disease [reviewed in Ref. (14)].

Chronic mold exposure at home, work, or school are associated with increased upper and lower respiratory symptoms (57). Commonly, the presence of specific IgE or positive SPT highlight an allergen-dependent pathway, but there is also evidence that mold is associated with respiratory symptoms, such as asthma in an allergen-independent manner. In fact, it has been stated that most common inflammatory reactions associated with fungi are non-allergic in nature (10, 20). Further, there is some evidence of a higher susceptibility to infectious disease with wheezing and non-wheezing lower respiratory tract illness, such as pneumonia, croup, bronchitis, or bronchiolitis (58). As chronic mold exposure is able to alter the immune response and could adversely affect the ability to combat infections (10), the trade-off model would suggest an adjustment of investment of costs of the immune system (59).

Up to 24% of the population has IgE antibodies or positive skin tests to common inhalant molds (55, 60, 61). Furthermore, when analyzing a cohort of subjects with respiratory diseases, the prevalence of sensitization is around 19% (61) or up to 44% among atopics (55). The incidence of asthma, and also the severity, has repeatedly been shown to be higher in patients with skin sensitivity to common indoor fungi [reviewed in Ref. (62)]. Mold exposure as well as dampness has been associated with new onset of asthma (63, 64). Mold allergy and mold-associated respiratory disease have been shown to be linked with both outdoor and indoor exposures (9). Meta-analyses have shown building dampness and mold to be associated with approximately 30–50% increases in various respiratory and asthma-related health outcomes (65). There are also other immunologic diseases produced by mold, such as hypersensitivity pneumonitis, but due to the paucity of generally available biomarkers, a significant emphasis has been placed mainly on type I allergy and asthma and less emphasis has been placed on other immunopathological mechanisms involved in the pathogenesis of type II–IV allergy (66).

Another issue is that mold exposure may increase sensitivity to commonly inhaled microorganisms and inert substances and increase the risk of secondary infections (67).

Respiratory symptoms in asthma include coughing, wheezing, chest tightness, and shortness of breath. Bronchial inflammation induced by allergic and non-allergic mechanisms produces narrowing of the lower airways producing the symptoms. As has been said above, this mechanism could have an adaptive origin. Bronchial asthma has been the focus of several evolutionary analyses. These are mainly based on a rapidly changing environment and the inflammatory potential of asthma is explained as an exaggerated immunopathological response in the context of the hygiene hypothesis. It states an increase of chronic inflammatory and autoimmune disease, as a consequence of a diminished exposure to infectious agents, symbiotic microorganisms, or parasites, which would be necessary for a balance between pro- and anti-inflammatory forces (68). In the special case of allergy and IgE production, an evolutionary perspective gives emphasis to a long-lasting history of mammals with IgE-inducing multicellular parasites, where chronic infections or stimuli would otherwise downregulate damaging effector response (69). Lappé proposed the asthmatic response to be a defensive mechanism to avoid dangerous mold to gain entry to the lungs. The risk of inhaling moist spores would be the selective force in the development of asthma (40).

As health effects of mold and dampness on the respiratory tract have been evidenced for both allergic and non-allergic patients, one possibility is that sensitization is only a marker and a special case of an ongoing inflammatory response against mold or fungi in genetically susceptible patients. Inflammatory and broncho-obstructive symptoms obtained by exposure to organic dust also produce similar symptoms. Norn concluded that the exposure to fungal spores enhances the histamine release triggered by both allergic and non-immunologic mechanisms (70, 71). Therefore toxic, rather than allergic or similar inflammatory, processes were involved (72).

Chronic fungal infection of the respiratory tract can affect immunocompromised or immunocompetent patients. There is increasing awareness of chronic fungal rhinosinusitis occurring in otherwise healthy hosts (73). Symptoms are not only due to local affection, but there are also reports of generalized symptoms, such as that in the case of neuro-muscular symptoms after infection by several molds and even local trichothecene production (74). Frequently there is a previous exposure history to water-damaged buildings.

Interestingly, sarcoidosis, a chronic inflammatory pulmonary disease of yet unknown origin has been associated not only with exposure to fungi, such as in mold-infested buildings, but *in vitro* studies also showed the implication of immune parameters typically associated with fungal contact, such as dectin-1, TLR2, and TLR4 (1, 75–77). This leads to the hypothesis that sarcoidosis could be due to an exaggerated inflammatory reaction in genetically susceptible individuals after mold exposure (75).

Thus, by this perspective, respiratory symptoms could be interpreted as a frontline defense against potentially invasive fungi and related conditions associated with mold toxicity. Symptoms in sick building syndrome may be induced by the total biological load in inhaled air through a combination of toxic and immunological responses (78), and this idea can be applied for DMHS.

## Extra-Respiratory Symptoms in DMHS

For symptoms associated with mold or mold-associated environment, different partially overlapping syndromes have been proposed. But mold is one of the possible causes in sick building syndrome. The WHO has classified the reported symptoms into broad categories, including: mucous membrane irritation (eye, nose, and throat irritation), neurotoxic effects (headaches, fatigue, and irritability), asthma and asthma-like symptoms (chest tightness and wheezing), skin dryness and irritation, gastrointestinal complaints, and more (79). The definition of mixed mold mycotoxicosis has been suggested for a wide range of multiorgan symptoms, including upper and lower respiratory symptoms, headache, dizziness, visual changes, cognitive impairment, or emotional dysregulation in patients with a history of exposure to mixed colonies of molds and their associated mycotoxins (80).

In our analysis, we can divide extra-respiratory symptoms into two broad categories: the first includes symptoms that are produced by an inflammatory reaction and the second can be seen as neurobehavioral.

Like in autoimmune disease, systemic inflammation can affect different organs, and therefore several descriptions of DMHS-associated disease include rheumatic or neurological symptoms (81, 82). The term *chronic inflammatory response syndrome* has been coined by Shoemaker et al. for multiorgan symptoms following exposure to the interior environment of a water-damaged building with resident toxigenic organisms, and their associated compounds (83). Otherwise, the interpretation of appearance of autoimmune disease in a modern environment with an over-reacting immune system and eventually exaggerated or unnecessary symptoms is similar to the application of the hygiene hypothesis in the field of allergy (68).

Likewise, DMHS can include symptoms in the field of psychology or psychiatry. In a study involving patients with exposure to mixed colonies of mold and their associated mycotoxins, stress-induced disturbances ranged from depression, affective loss of control, to cognitive disorders (80). Memory loss, headache, fatigue, balance loss, or cognitive dysfunction is also complained by patients with mold-associated sick building syndrome (51) and holds also for molds as the postulated cause. Thrasher et al. reported on a family, whose five members developed symptoms consistent with myalgic encephalitis–chronic fatigue syndrome after exposure to molds and mycotoxins in a water-damaged home (84).

Interestingly, the term “behavioral immune system” has been coined having triggering psychological responses that are adaptive for disease avoidance (85). Infection-avoidance behavior concerns actions taken by an animal to reduce its chances of becoming infected with pathogens or parasites. In fact, symptoms such as sneezing, diarrhea, and emesis are not only induced by immune reaction, as they can also be interpreted as a behavioral response (50). Symptoms are thus a response to environmental signals associated with some danger and can be seen as a system to motivate escape from and avoidance of situations that harm fitness (52).

The presence of multiorgan symptoms, which are frequently unspecific, and the fact that cause and health effect relationships is difficult to assess or prove, can now be assessed with a different perspective, where exaggerated or apparently unnecessary symptoms can help the patient to avoid potentially damaging environments.

## Conclusion

The presented evolutionary analysis of DMHS shows evidence that the relationship between fungi, mold, and humans has provided us over eons of time with important defense mechanisms, without these we would not have survived as a species. Innate and adaptive immune defense mechanisms as well as a neuroendocrine system aimed at avoiding contact with potentially harmful fungi have a great interindividual variability, but display a mainly pro-inflammatory or exaggerated potential in those patients suffering from DMHS.

I show some evidence that symptoms and disease in DMHS are associated with an elevated inflammatory status, but the observed respiratory and extra-respiratory syndromes are elicited by different mechanisms, ranging from IgE-mediated allergy to multisystem, autoimmune phenomena, as well as neurobehavioral components. Inflammatory markers have already been associated with fungal exposure, but the association can be two sided. It seems plausible to think that mold exposure induces an inflammatory reaction, but it could also be the other way round, in that genetically susceptible individuals with a higher pro-inflammatory profile due to their evolutionary past, will respond to lower threshold of mold and dampness exposure. Like women being generally more prone to most autoimmune diseases as a trade-off to a higher inflammatory potential to combat infectious disease, women do not only suffer more often form sick building syndrome but also display higher mold-specific IgG-antibodies than men (86, 87). If the hypothesis is true that MHSD is a trade-off to avoid or defend potential invasive or dangerous pathogens, these individuals should have a lower probability of invasive fungal disease. As has been said, fungal systemic infection in humans is rare, but future epidemiologic studies could evaluate those patients, who are at risk to acquire fungal disease and search not only for an exposure history but also for symptoms or lack of symptoms associated with DMHS.

On the other side, there is a small number of fungi that are able to colonize without infection in humans, such as *Aspergillus* or to persist in a latent state, others behave with lifelong dormant infections, such as *Cryptococcus* or *Histoplasma capsulatum* (37). With the increasing availability of biomarkers for exposure to molds or mold components, such as mycotoxins, but also for presence of fungi in human samples, clinical studies could now assess the frequency of clinical but also subclinical colonization with these fungi in patients with a history of DMHS compared to those with similar exposure history and no evidence of fungal colonization. In this case, if symptoms in patients with DMHS have an ongoing adaptive value, these patients should have a lower frequency of colonization or latent infection.

What is clear is that adverse health effects of mold exposure should prompt not only a search for the cause, but mainly lead to improve indoor environment quality, which will potentially benefit many more people than the individual identified with these health claims.

## Author Contributions

The author confirms being the sole contributor of this work and approved it for publication.

## Conflict of Interest Statement

The author declares that the research was conducted in the absence of any commercial or financial relationships that could be construed as a potential conflict of interest.
